# Policy makers’ perspective on the provision of maternal health services via mobile health clinics in Tanzania—Findings from key informant interviews

**DOI:** 10.1371/journal.pone.0203588

**Published:** 2018-09-07

**Authors:** Nyasule Majura Neke, Gema Gadau, Jürgen Wasem

**Affiliations:** 1 National Institute for Medical Research, Mwanza Centre, Tanzania; 2 Institute for Health Care Management and Research, University of Duisburg-Essen, Essen, Germany; 3 Plan International (Kisarawe Office), Dar es Salaam, Tanzania; TNO, NETHERLANDS

## Abstract

**Objective:**

To explore the operational feasibility of using mobile health clinics to reach the chronically underserved population with maternal and child health (MCH) services in Tanzania.

**Design:**

We conducted fifteen key informant interviews (KIIs) with policy makers and district health officials to explore issues related to mobile health clinic implementation and their perceived impact.

**Main results:**

Policy makers’ perspective indicates that mobile health clinics have improved coverage of essential maternal and child health interventions; however, they face financial, human resource-related and logistic constraints. Reported are the increased engagement of the community and awareness of the importance of MCH services, which is believed to have a positive effect on uptake of services. Key informants (KIs)’ perceptions and opinions were generally in favour of the mobile clinics, with few cautioning on their potential to provide care in a manner that promotes a continuum of care. Immunization, antenatal care, postnatal care and growth monitoring all seem to be successfully implemented in this mode of service delivery. Nevertheless, all informants perceive mobile clinics as a resource intensive yet unavoidable mode of service delivery given the current situation of having women and children residing in remote settings.

**Conclusion:**

While the government shows the clear motive, the need and the willingness to continue providing services in this mode, the plan to sustain them is still a puzzle. We argue that the continuing need for these services should go hand in hand with proper planning and resource mobilization to ensure that they are being implemented holistically and to promote the provision of quality services and continuity of care. Plans to evaluate their costs and effectiveness are crucial, and that will require the collection of relevant health information including outcome data to allow sound evaluations to take place.

## Introduction

Accessibility of maternal and child health (MCH) services continues to be an intractable problem in the developing world. To date, many children under five years of age, including newborns and their mothers, still have no access to essential life-saving medical interventions[1]. Evidence suggests positive associations between access to essential maternal and child health interventions and improved child and maternal health outcomes, respectively [2–5]. However, in a poor resource setting access to such services is very limited[6] and the risk of death following pregnancy and childbirth is high[2, 3].

Tanzania, which is a predominantly rural, low-income country in the eastern part of Africa, has a maternal mortality ratio of 556 per 100000 live births and a mortality rate among children under five of 67 deaths per 1,000 live births. Some of these deaths are preventable, yet to date, pregnant women and children in Tanzania still lack access to life-saving maternal, and child live health interventions. A large proportion of those children and pregnant women who lack access to care with an increased risk of maternal and under-five mortality live in remote, hard to reach settings[7]. Trends from the recent demographic health survey indicate that urban women are more than twice as likely to receive ANC from health personnel as rural women. Vaccination coverage in children under five is higher in urban areas as compared to rural areas, and seven out of ten urban children were taken to the health facility when sick, while less than half of the rural children received treatment or advice from a skilled health provider[8]. Women in Tanzania and similar low-income settings frequently identified multiple barriers preventing them from accessing health care for themselves or for their children, which includes the distance from their village to the hospital[6–8].

For the past four decades, Tanzania has been implementing and adopting different strategies aiming at improving MCH services utilization. However, utilization has remained at a level that is not satisfactory and declining in some areas due to many factors, including a limited number of health facilities and long travelling distances from health facilities to the villages[7, 8]. To overcome these difficulties, a variety of measures has been developed to improve access to and utilization of MCH services [9]. These include, but are not limited to the use of community health providers, use of mothers as peer educators, mobile health clinics and outreach programs–often using a mobile van or bus, and sometimes conducting home visits.

The use of mobile health clinics in Tanzania dates back to the 70s. The Ministry of Health and Social Welfare (MoHSW) through the Council Health Management Team (CHMT) in collaboration with development partners use mobile health clinics to offer reproductive, maternal and child health services routinely throughout the year[10]. Ideally, the mobile vans with health providers and a driver visit each village once per month and offer MCH services. They target populations residing seven kilometres away from the nearest health facilities and villages that do have health facilities but cannot provide those services[11]. The mobile clinics have approximately 4–5 health providers in one team, while only one health provider does outreach services. A wide range of MCH services are provided in mobile clinics including immunization, family planning, antenatal care, child growth monitoring and HIV/AIDS interventions like PMTCT services, counseling and testing. Provision of MCH services through mobile clinics and outreach services has been reported to improve the geographical accessibility of health services [11–13]. For instances during the reporting year of 2016/2017, mobile services in Tanzania reached over 500,000 women with family planning services[12]. In the Kisarawe district in Tanzania alone, mobile clinics reached over 20,000 children under five with essential health interventions including vaccination in 2016. In the same district, more than 540 pregnant women and over 292 post-natal women were reached with essential medical care. [11].

Despite their usefulness, documentation of different aspects of this mode of service delivery remains patchy in Tanzania. Few reports exist that have described how these services are provided [10, 12, 13]; however, they are primarily based on family planning. Understanding that health service provision is context specific, it is worthwhile to explore and document operational feasibility and perceived impact of this mode of service delivery in the context of maternal and child health services. Our objectives were to use a qualitative approach to explore the operational feasibility and perceived impact of mobile health clinics providing MCH services in remote areas of Tanzania. Conducting a qualitative study is essential to better understand the essential aspects of the implementation of this model of service delivery.

## Material and methodology

### Study area and population

The fieldwork was conducted in Kisarawe. Kisarawe is one of the six districts of the Pwani region of Tanzania. The district has a population of 101,598 out of which 50,967 were women of reproductive age as estimated by the 2012 National census[8]. It has four administrative divisions, namely, Mzenga, Chole, Sungwi and Maneromango. In these divisions, there are a total of 15 wards with 76 registered villages and 226 hamlets. Kisarawe was identified as an appropriate area for this study because of its low vaccination coverage among children under five years of age and low utilisation of antenatal care among pregnant women. It is estimated that only 34% of pregnant women attend ANC to their fourth visit and 65–75% of children under five received essential vaccination[14].

### The study design and questions

This was a case study that utilized qualitative methods. This design is useful for the understanding of the detailed situation surrounding the provision of MCH services through mobile clinics in Tanzania[15]. This study also followed the consolidated criteria for reporting qualitative research (COREQ)[16] and the dully filled checklist is provided as supplementary documents(S1 File). The study aimed at answering the following questions:

1. How do policy makers perceive mobile health clinics?
2. What are the perceived impacts of using mobile health clinics?
3. What are the challenges facing implementation of mobile health clinic activities? And what are existing opportunities?

### Sampling and sample size

We selected a total of eighteen KIs using purposive sampling methods. We included five participants from the Safe Motherhood Unit of the Reproductive and Child Health Department at the MoHSW, five members of the CHMT, three health providers who are part of a team providing services at a mobile health clinic, and three informants from the international non-governmental organizations providing MCH services through mobile clinics. We first contacted the Safe Motherhood Unit of the MoHSW, which provided us with the organizational structure of the service provision. We later on selected the informants according to their role and responsibility in the service provision. In the end, we managed to interview 15 out of the 18 KIs we selected.

### Description of the study participants

The criteria for selecting the KIs were based on three factors: Informant experience and involvement in the subject area, institutional and professional reputation in issues related to MCH services provision. Our informants from the MoHSW were senior officials from the Ministry of Health with a background in obstetrics and gynecology (3). At the council levels, there was a clinician (1); a pharmacist (1); health planners (2); nurses (3) and health officers (3); while at the international organization there were social scientists (2) and a clinician (1). Apart from the two social scientists and one health officer who had a work experience of 6 to 10 years, all other participants had more than ten years of experience in the area of MCH services.

### Data collection methods

KI interviews were conducted by two researchers (NN and GG) as face-to-face encounters at the offices of the KIs. Only the interviewers and the KI were available during the interviews. 12 out of 15 KIs accepted to be digitally recorded during the interview while the other three were not comfortable to be recorded; hence their interviews were noted on shorthand and notes expanded immediately after the interview. Recorded interviews lasted around 25–45 minutes. Interviews were conducted in Swahili, guided by a pre-tested, semi-structured interview guide (S2 File and S3 File). The guide was pre-tested on five participants and fine-tuned accordingly. During the discussion, the flow of discussion varied from one informant to the other, depending on the way the discussion unfolded.

### Data management and analysis

**Fig 1:**
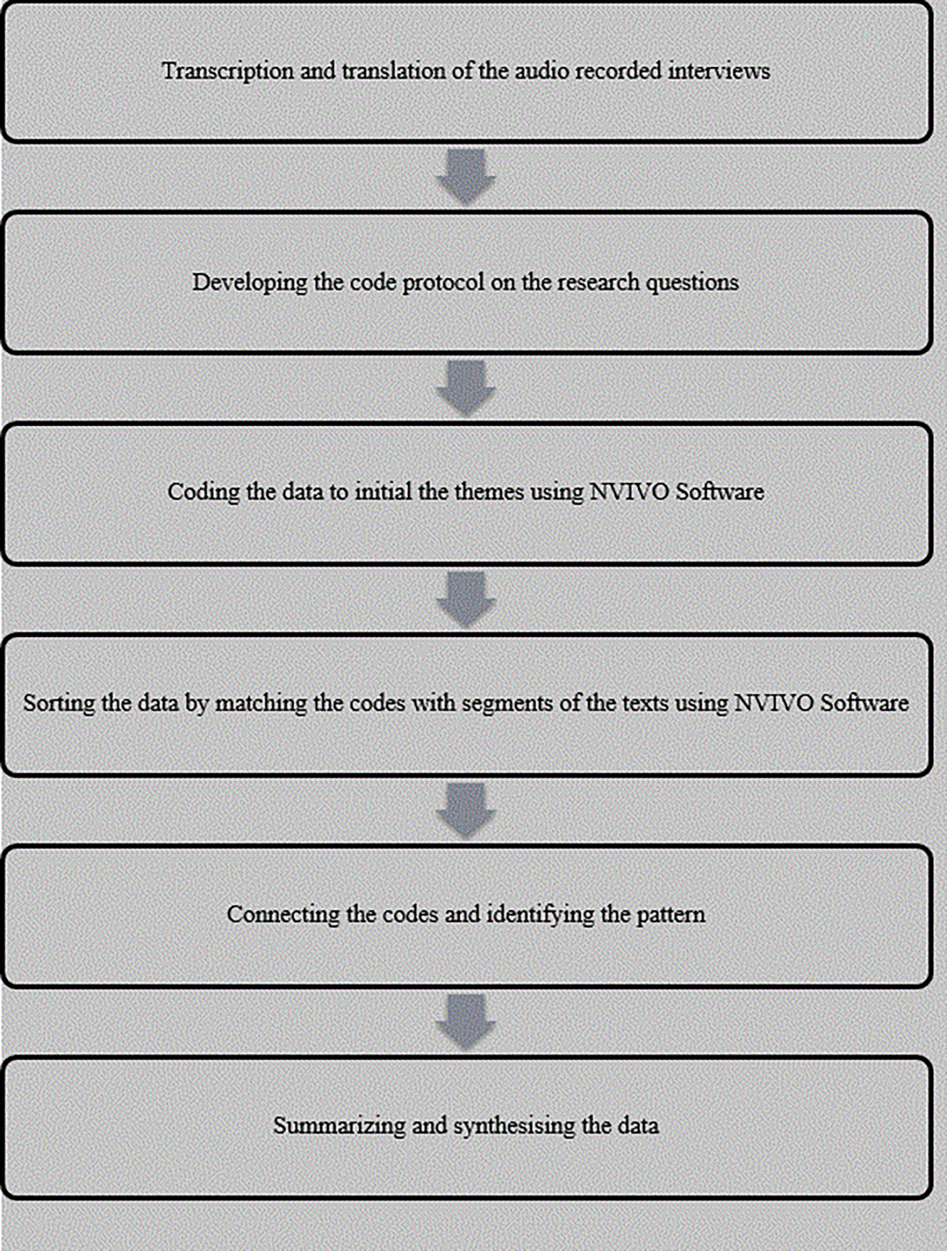
Steps followed during data management and analysis.

The audio recorded data was transcribed by an independent professional transcriber following the standardized transcription protocol as recommended by qualitative methods guidelines[16, 17] and following the steps depicted in (Fig 1). One translator translated the Swahili transcript into English, and then two research scientists (NN and GG) read the transcript and made edits. The English versions of the transcripts were then uploaded into NVivo Software QRS 2011. We used the thematic content approach[15, 18] in which we read each transcript carefully and repeatedly to identify relevant text.

After that, we developed a data-coding scheme by organizing similar codes into categories and used it to code all the data. We read the quotations that were attached to the codes and summarized the main description in the memos. In the initial stage one researcher (NN) independently conducted the open coding thematic content analysis to identify themes and concepts. Then two researchers performed coding simultaneously. In the end, the three researchers (NN, GG and JW) validated the codes and selected the final codes. Although one researcher performed the initial thematic content analysis, the continuous consultative approach offered reflexivity and explored relationships between these themes and concepts.

### Ethical considerations

The study was granted ethical approval by the National Research Ethics committee in Tanzania (Ref. No. NIMR/HQ/R.8a/Vol. IX/2061) and the Ethics Committees of the Faculty of Medicine of the University of Duisburg-Essen in Germany (Ref. No. 15-6512-BO). We obtained a signed informed consent from all KIs. All our KIs could read and write, so they signed the consent form after reading and understanding the aim of the study.

## Results

We conducted fifteen KIIs and identified five themes as follows:

- Perception towards providing services using mobile health clinics
- Perceived impacts of using mobile clinics to support MCH
- Strategies used to increase uptake of MCH services at the mobile clinics
- Challenges facing mobile clinics operations
- Guideline use in the provision of services in mobile clinics

### Perception towards providing service using mobile health clinics

During their account of this approach (providing MCH via mobile health clinics), respondents supported the idea because it increases the coverage of interventions and reaches remote areas that do not have health facilities. When asked about the policy of “*each village one dispensary*”, they argued that it would take long for each village to have a dispensary and therefore, mobile clinics are significant. One participant responded:

“*Some people live in unimaginably remote areas*, *others migrate from one place to another*, *and others refuse to vacate from those remote areas… we understand that we [the country]*, *will achieve the goal of having a dispensary in each village but in the meantime*, *because they [people] also need services*, *we are sending those services through mobile clinics”*. (Medical Specialist, KI #1)

However, they acknowledged that services should be provided in a manner that will promote the continuum of care. It is for that reason that they have long ago advised the councils to establish maternity homes in which pregnant women will live to wait for their time of delivery. These homes are close to the health facilities, so women who are attending antenatal care at the mobile clinics can live there when the time of delivery is approaching.

*“…The Ministry of Health recommends that each facility that provides delivery services should have what we call maternity waiting homes or a house of waiting…*.” (Medical Specialist, KI #8)

These homes are already available and operating in some council. Councils are the ones responsible for managing these homes in collaboration with the clients. The clients are responsible for buying their food while they are there, and the council covers electricity and water bills. The respondents feel that these homes provide an opportunity for all those women who live too far away from the facilities to deliver their babies in the hospital.

Different opinions emerged regarding the practicability of providing services in the mobile vans, especially on issues related to privacy and confidentiality:

‘…*In that car*, *there is a space like a consultation room; there is a bed and a chair*. *You can sit inside with a woman [client]*, *and you can provide services to her without people outside knowing what is being discussed or done in that room*. *Alternatively*, *sometimes we go to the office of the village secretariat or a school*, *and they provide a room that we can use for that day*. *Therefore*, *these services are provided in a manner that ensures client confidentiality and privacy is adhered to…”* (Nurse, KI #10)

On the other hand, a senior official at the international NGO stated:

‘… *You must also consider clients’ privacy and availability of enough space for clients to rest when they need to*. *The mobile van cannot offer services in an environment where clients can be comfortable and at the level of privacy indicated in guidelines of health care delivery* …’ (Clinician, KI #15)

Despite different opinions about privacy and confidentiality, there was a general recognition that mobile clinic activities need to be improved. Highlighted was the importance of ensuring that medical supplies and laboratory equipment are sufficient to allow them to offer standardized care and to make services more attractive.

‘.. *If we have simple and easy to carry laboratory machines for simple but very significant investigations like MRTD [Malaria Rapid Diagnostic Test] for Malaria*, *hemoglobin testing*, *urinalysis for UTI and protein in urine*, *our services [at the mobile health clinics] will be much better and more attractive…*’(Nurse, KI #10)

The issue of incentive came up in all interviews. Senior officials both at the Ministry, Council and International NGO insisted on having a plan to reimburse the health workers who travel to provide care in the mobile van. Reason for that being the fact that these health workers sometimes find themselves stuck in the village and they have to spend a night there in case the car breaks down or if there are too many clients. Hence they felt that giving them something small to compensate for the hours that they work overtime would motivate them.

‘…*Health providers are motivated because of the incentives that they receive*, *and they can take the risk of going there [to those remote areas]*, *and*, *even sometimes sleeping over there to provide services…*’ (Social Scientist, KI #13)

Coinciding with those views were the statements from the health providers, although they did not attach too much importance to incentives; instead, they insisted more on the availability of tools to work with.

*‘…*. *For us*, *it remains to be our responsibility*, *and it does not matter whether or not we receive incentives or money […*.*] although in the past we used to get an incentive when we returned from providing services*. *That was thoughtful*. *However*, *even now that we do not receive the incentive*, *we still see this [going for mobile outreach services] as our responsibility… It is the same job that I would have done here [at the static clinic] if I will not go to the field*, *so it is not a problem…'* (Nurse, KI #11)

Different views arose when they were asked which MCH interventions should be ideally provided at the mobile clinics. Few (2) said that mobile clinics are ideal for interventions like family planning, immunization, growth monitoring, HIV counseling and testing, and health education specific for MCH interventions. The majority of the respondents said that all MCH interventions could be delivered at the mobile clinics in an integrated manner, which means women and children can come and receive more than one intervention at once and reduce distances travelled by the clients.

Specifically for antenatal care service provision, respondents gave different perceptions of mobile clinics providing MCH services. Few (2) were skeptical about the whole situation of using mobile clinics to provide MCH services, arguing that they do not uphold the principle of a continuum of care. A respondent from the MoHSW said:

“[The concept of] *Mobile clinics is a good idea because it sends services where the demand is*, *but it will not be able to provide services in a manner that will fulfill the continuum of care…*.*"* (Medical Specialist, KI #1)

The identified lack of health care facilities closer to where people live as recommended by the national policy is the main reason that made participants share a common positive perception towards using mobile health clinics. Yet, participants shared their reservations in some aspects including and not limited to availability of human resources, how services are provided and the aspect of continuity of care.

### Perceived impact of using mobile clinics to support the provision of MCH services

The perceived impact of mobile clinics regarding increasing coverage of MCH interventions came prominently during the discussions. All respondents reported that mobile clinics had changed the picture of the coverage of all MCH interventions. One respondent reported that mobile clinics had increased the coverage of immunization of all antigens in a significant amount.

“…*The immunization coverage was very low; for example*, *pneumococcal conjugate vaccine [PCV] coverage was only 70% and in other antigens only 60%*. *Oral polio vaccine [OPV] was the only antigen that had coverage of 80%*, *and this is also low since the country’s target for all antigens is supposed to reach 90% and above* …” (Health Officer, KI #3).

Another informant, who works specifically in family planning interventions, added how mobile clinics have contributed to the reduction of maternal and child death. He highlighted that:

*“…*. *Through mobile clinics we have managed to increase the CYP [Couple Years of Protection]; that increase is also consistent with the number of clients who utilize our services* …*…*. *that shows us that we have a huge contribution in the provision of family planning services of all methods in the nation*..*…” (*Clinician, KI #15)

Participants also suggested that mobile clinics have raised awareness of the importance of MCH among the communities that they visit. Both supervisors and the frontline health providers agreed that the level of awareness that is seen now is a result of mobile clinics activities that are sent to their village. One informant narrated this kind of impact using the following example:

*“…For instance*, *when it happens that we cancel the visit*, *villagers and women will ask why we have not visited them*, *and when we will go again*. *They will call our mobile clinic number to inquire on when we return*. *That indicates that the people now understand the importance of vaccination and antenatal care…”* (Nurse, KI #5)

On their account on awareness informants implied that the ongoing mobile health clinic activities have to some extent broken the barrier of men not wanting anything to do with their wives' and childrens’ health. They also suggested that men are now seen more often escorting their wives to the clinics or even bringing their children for vaccination—even in areas where that was previously impossible. An informant explained:

“…*The number of men participating in the mother and child health issues has also increased as compared to the past…moreover*, *we see quite some men now escorting their partners to the clinic*, *and others they bring their children to get vaccinated"*. (Social Scientist, KI #7)

The perceived impacts narrated by the participants were predominantly based on the number of people reached with the interventions. It also consists of aspects of reaching people with the information regarding available interventions. Since the impact of maternal and child health services were described as numbers of people reached or utilized the intervention, minimal attention was given to matters relating to the quality of those services.

### Strategies used to increase uptake of MCH services at the mobile clinics

In their description of approaches adopted to increase uptake of services at the mobile health clinics, informants reported using a national strategic plan for health promotion[19]. Adopting this plan, they partner with villagers and their leaders to improve utilization of maternal and child health services in the villages they visit. The majority of the participants stressed the importance of health education as a strategy towards improving uptake of services. For example, one participant narrated:

“…*We created user-friendly learning materials which contain information about the interventions*, *and we talk to community members and share with them the importance of the available essential interventions*…” (Health Officer, KI #12)

Use of adverts is also mentioned as a possible way to spread the news about the availability of services in the villages. An informant described this by saying:

*“… We send advertisements in collaboration with the districts’ and councils’ authorities that we want to provide services*. *In other areas*, *we use messengers*, *in which a person is hired to pass through the streets with the microphone in the car*, *and announce that we will be providing reproductive health services together with family planning at a certain place and on a certain date…” (*Clinician, KI #15)

Regarding the question of which to use in sending the information to the people, all informants acknowledged that influential community members are the best ones to use. These are perceived to understand their communities more hence making it easy for them to reach the people with information. An informant said:

“…*We engage influential people who are in those villages*. *Those people are the village health workers and the village leaders*. *That is the first strategy which we use*, *those people are near to the communities we want to reach*, *so we communicate with them*, *and then they share the news with the community…*” (Nurse, KI #11)

The use of community mobilization as a strategy to increase the demand for these services was influenced by the shared idea and existing evidence that better utilization of maternal and child health care services depends on mobilizing the entire community. In participants’ framework of demand creation, reaching to key opinion leaders in the communities and sending the information regarding the availability of services through them was a key component of promoting these services.

### Challenges facing delivery of MCH services at the mobile clinics

In their account, policy makers indicated that resources concerning funding and human resource are the major challenges facing the mobile clinic services. One informant said:

“…*Mobile and outreach services are very expensive*. *I cannot provide you with exact costs in numbers*, *but these services are expensive because of many factors*. *You must pay for the car and maintain it; you also have to take into account the costs of supplies and awareness campaigns* …” (Clinician, KI #15)

Despite the cost implications, some informants cautioned that the concept of mobile clinics is something that the country cannot merely terminate due to many reasons. One being that maternal death should not be allowed to happen at any cost. Describing this, a senior official at the MoHSW said:

*“…I normally tell people to improve health care services*, *you need heavy investment*, *and the outcome will be seen later… also*, *now try to look at the life of a woman*, *when she dies because of not receiving these interventions*, *how costly it is there… so if you see that going for outreach services is expensive*, *then try maternal death*, *and you will know what is costly*..*” (Medical Specialist*, KI #8)

They instead recommend and advice that councils should make a sound and sustainable plan of reaching these women and children since they believe it is not an issue of funding, but rather a priority setting problem. One official explained:

*"*. . . . *They should add it on the CCHP so that it can be something that must be done given its significance*, *and it has a play in improving maternal and child health* …*”* (Health officer, KI #6)

On the other hand, informants working with the development partners said that the council’s health department had assured them that these services would continue, and plans to mobilize funds are already in place.

“… *We had a meeting with DMO and CHMT*, *they have assured us that the activities of this project are in the budget and they have shared with us that budget; therefore the issue is to execute that budget and to implement as it was planned* …” (Social Scientist, KI #7)

Conversely, informants from the council agreed that these activities are included in the Council Comprehensive Health Plans (CCHP). Yet, in many cases, the activities get into the plan either in a piecemeal or not in time hence rendered it difficult to be executed as expected;

*"…You might convince them*, *and it can get into the plan*, *but it will not happen in the way it was requested…*” (Health Officer, KI #12)

When confirming this with the member of the CHMT, they agreed that they had made plans to ensure that MCH services continue in villages where there is no hospital, but they are always caught up in the situation that they receive less money compared to what they have budgeted for.

*“…We have tried to incorporate mobile clinics and outreach services into our health plans*, *but even if you put them in that health plan*, *the challenge is that we have minimal budget*, *for example*, *the year before last year we had 275 million for the whole district*, *last year the budget was reduced from 275 to 250 million…*. *and this year it was reduced from 250 to about 205 million*. *Therefore*, *each year the budget decreases and the prices for equipment and supplies do increase…” (*Council Official, KI #9)

Additionally, mobile clinics were said by all respondents to create a shortage of health providers in the facilities. Demonstrating this, one respondent said:

“…*Because whenever we take a health worker from one facility to the mobile clinic*, *it means that a particular facility will have a shortage from that outsourcing*…” (Medical Specialist, KI #8)

Finally, the impact on the quality of care when health workers carry out additional tasks like collecting medical information during mobile clinics activities was raised by a number of KIs including a senior MoHSW representative:

*"…Collecting information is not very hard if you have time and enough human resources… Moreover*, *if we are going to rely on the same person who is providing the service and then collects the information*, *we will be either compromising the quality of the services or the data collected…” (Medical Specialist*, *KI* #14)

Policy makers and implementers used a multi-sectorial lens to construct their views regarding the challenges facing mobile health clinics. Using this approach, it was clear that hurdles impending mobile health services are crosscutting ranging from poor road infrastructure, insufficient funding, shortage of medical supplies and human resources. These challenges together are said to threaten not only the sustainability of these services but also the provision of quality of care.

### Guideline use in the provision of services in mobile clinics

Respondents were aware of the available guidelines for the provision of MCH services. These include guidelines related to antenatal care provision, HIV care and treatment, as well as integrated management for childhood illness[20–22]. They were also able to describe key aspects that are in those guidelines and affirmed their availability at each mobile clinic. However, the mere fact that these guidelines existed did not mean that they were used as desired because of existing barriers. For instance, shortage of resources—both human and supplies—was mentioned to be among the barricades that hinder guidelines implementation. A respondent described that:

“…*The team that goes to the field normally is incomplete because of the shortage of staff*, *and also because we cannot leave the health facility without health providers*. *In many cases you may find that the clinical officer and the health officers are missing…*. *(*Health Officer, KI #6)

So that makes them provide services using the number of health workers they have, even if this does not comply with the guidelines. Describing what the ideal service provision at the mobile clinic should be, one informant illustrated that the team should be complete for all services to be provided within standards. Lack of reagent and other medical supplies was also another obstacle to providing services according to the guidelines. A senior nurse at the council pointed it out by saying that:

“…*Guideline for the provision of antenatal care*, *for instance*, *states that women should be screened for STI infections like syphilis and HIV*, *but that is not always happening because of lack of reagen*t…” (Nurse, KI #5)

Those barriers are known from all levels of the health systems. For example, a respondent from the ministry level which is responsible for guideline formulation and translation echoed similar concern as those raised by the respondent at the council level:

“…*in many instances*, *we find ourselves in the situation where we do not have needed supplies and equipment that they can use as recommended in the guidelines and according to how we trained them […] Instead they end up improvising using what is available and not what is recommended …*” (Health Officer, KI #12)

It goes without saying that the guideline use sits at the crossroads. On the one hand, the government trains health providers and promotes utilization of clinical guidelines when providing health care. Yet lack of essential health care equipment and supplies limit the overall objective of providing care according to standardized recommendations.

## Discussion

The current study used qualitative methods to explore operational feasibility and applicability of using mobile health clinics to provide maternal and child health interventions from the policymakers’ perspective. The result indicated that policymakers support the mobile health clinics approach because it presents an opportunity to improve access to MCH services to the disenfranchised population. The discussion, however, suggests the existence of challenges that need to be addressed to improve not only the quality of care, but also to ensure sustainability of these services. The results provide evidence that supports the mobile health clinic approach in areas that lack health care. To some extent, our results complement those obtained previously in Tanzania[10, 12] by documenting relevant operational issues on providing maternal and child health interventions via mobile health clinics.

A clear majority of key informants attested that mobile health clinics enabled women and children who live in remote, hard to reach areas to access life-saving interventions. This is in line with the studies that have been done in other settings in both developed and developing countries [23–25]. In those studies, it is reported that the mobile health clinics have the potential to increase early access to MCH interventions. This general support met skepticism when antenatal care services were concerned due to the argument that the model does not uphold principles of a continuum of care. Noted is the possibility of linking ANC services provided at the mobile clinics with the existing waiting homes to bridge the gap and ensuring that these women deliver at a health facility. Studies that investigated the impact of mobile health vans specifically for antenatal care affirmed that mobile health vans enabled women living in remote, underserved populations to start ANC during their first trimester [9, 24–26]. Nevertheless, the authors concluded that there is a need to explore this further to improve these services.

Despite the general views in favour of mobile health clinics, nearly all of the key informants acknowledged that mobile health clinics are resource intensive, both in terms of supplies and time. There was also a consensus that extensive amounts of time needed during travelling to remote places as a result of bad road infrastructure and scarcity of resources are significant barriers facing mobile clinics operations. Researchers who evaluated mobile clinics providing immunization services[23, 27], family planning[10, 12, 13] and other curative health care[28] affirmed that this mode of services delivery requires a large number of resources in terms of fuel and vehicle maintenance. On the contrary, albeit few, studies on cost and effectiveness of mobile health clinics reported that this mode of service delivery could provide a significant return on investment [13, 29].

Closely related to that, shortage of human resources hinders collection of essential health information during the mobile clinics’ activities. Our findings show that an overworked health provider would not regard recording health information as a priority when she or he has many clients to see and is alone at the mobile clinic. That may imply that health information collected in the mobile clinics may be of questionable quality and that it may be unreliable for planning. For instance, an evaluation from the mobile clinic in Malawi indicated that collection of health information at the mobile clinic was compromised because of a high volume of patients and few health providers, which makes health providers allocate their time in care provision, and ignore recording of health information[30]. Similar challenges were reported elsewhere, in which absence of patient outcome data due to lack of resources to gather information was seen as a fundamental challenge for mobile clinics [31].

Drawing on the wealth of data gathered from these voices, this work offers several recommendations to improve mobile health clinic services. Firstly, councils should consider allocating more resources in the area of maternal and child health, which will help services to be delivered in a manner that is according to standard procedures. Secondly, the government should ensure that portable and easy to use laboratory equipment is available in the mobile clinics. These recommendations are similar to those raised in previous studies, which suggested the need for reviewing and strengthening mobile health clinics and ensuring that resources are available [24, 28, 32].

Our findings emphasize the need for councils to ensure that they have good planning and adhere to budget allocations. This came about when respondents who are also members of the CHMT raised concerns that their plans are either not being approved according to how they see the situation or that they get approved in piecemeal, which prevents them from providing care smoothly. In line with that, our findings show that in many cases budgets are not allocated as they have been planned and often re-allocation of funds occurs. That poses a barrier to the implementation of activities, irrespective of those activities being in the plan. Other studies carried out in Tanzania have also found that district health plans are rarely implemented as planned, and lots of re-allocation and re-setting happens throughout the year [33]. It can therefore be argued that the councils will benefit from good planning to address those challenges, which seems to be rooted in poor or inadequate implementation of existing priority setting frameworks.

### Strengths and limitations

The study adopted qualitative methods, which allowed gathering detailed information on mobile health clinics. This approach brought forth a detailed account of what is happening in the country when mobile health clinics are concerned[34]. The study gathered information from informants who make decisions at the national level and council level, which limits the threats of bias in information between the two decisions making levels.

It is plausible that a number of limitations could have influenced the views expressed in this paper. Firstly, in depth interview methodologies are prone to recall bias because of incorrect memorization of some aspects under investigation. However so, this was minimized because the interviews were conducted while the mobile clinics activities were ongoing hence the likelihood of these KIs not to remember what was happening was low. Additionally, the two researchers who participated in the interview have an extensive expertise in the field, which was instrumental in conducting this study.

Secondly, this study brought together views from informants at the national level and one district council on mobile health services; however, it did not gather information from other councils in Tanzania. Therefore, these results should be treated with considerable caution because they may not be generalized in councils that are different from Kisarawe. A study that can include informants working in other councils will be useful to get a broader idea of these services in the country.

### Conclusion

This article has explored the reality of the implementation of mobile health clinics that support MCH services in Tanzania. While the government shows the clear motive, the need and the willingness to continue providing services in this mode, the plan to sustain them is still a puzzle. As this study has illustrated, these services face enormous challenges that threaten not only their sustainability but also their quality with no concrete and tangible plans from the government to overcome them. We argue that the continuing need for these services should go hand in hand with proper planning to ensure that they are being implemented in a holistic manner promoting the provision of quality services and continuity of care. Plans to evaluate their costs and effectiveness are crucial, and that will require the collection of valuable health information including outcome data to allow sound evaluations to take place.

## Supporting information

S1 File Consolidated criteria for reporting qualitative studies (COREQ): 32-item checklist. (DOCX)

S2 File Annex 1a semi-structured interview guide for policy makers. (DOCX)

S3 File Annex 1b semi-structured interview guide for implementers. (DOCX)

S4 File Annex 2 Key informants interviews information sheet and consent form. (DOCX)
