# Supplementary material for: Policy makers’ perspective on the provision of maternal health services via mobile health clinics in Tanzania—Findings from key informant interviews
Source: PLoS One. 2018 Sep 7;13(9):e0203588. doi: 10.1371/journal.pone.0203588 (PMC6128610; doi:10.1371/journal.pone.0203588)
Supplement: S2 File — (DOCX) [file pone.0203588.s002.docx]

**Annex 1a**

**Semi-Structured Interview guide for Policy Makers**

| **Questions** | **Answers** |
| --- | --- |
| **Maternal Health Services Policies and Guidelines** |  |
| Can you tell me about the government policy for the delivery of maternal health services?   - Can you summaries the key elements of the policy - Is the policy implemented? |  |
| Which donors and development partners are active in supporting maternal health services in the country? |  |
| What activities are currently being implemented to improve utilization of maternal health services?   - Mobile Health Clinics- Where? How often? Who is doing? Who is funding? - Community based interventions- Where? How often? Who is doing? Who is funding? |  |
| How successful to date has the effort to improve uptake of maternal health services in Tanzania especially for women living in remote areas?   - How success is measured? - What challenges have been encountered? |  |
| **Provision of Maternal Health Services (***I would now like to move on and discuss different platforms that are used to provide maternal health services in Tanzania***).** |  |
| Currently what maternal health services are being provided?   - Preventive services - Curative services? - How are they delivered? |  |
| What is the most used platform to deliver maternal health services? |  |
| What other service delivery model has the country considered to use to provide maternal health services? |  |
| Are there models of service provision specific for maternal health interventions? |  |
| Which delivery mode has been the most cost effective in relation to delivery of maternal health services? And why is it cost effective? |  |
| How do the ministry of health and social welfare ministry decide the way maternal health services should be delivered? |  |
| What is the working relationship between MOHSW and other stakeholders in relation to provision of maternal health services? |  |
| **Mobile Health Clinics strategy and implementation** (*I would like to move onto discussion about delivering health services through Mobile Health Clinics with you*) |  |
| Have you ever heard about the mobile health clinics?   - What do you know about it? How many types of mobile health clinics exists |  |
| Are you aware of any mobile clinics currently being implemented in the country?   - If yes, where, and who is implementing them, and why are they implementing them in those areas - Which services are being provided in those mobile clinics |  |
| Did you know that there have been some small projects that deliver maternal health services through mobile health clinics?   - Tell what you have heard (Marie Stopes, Plan International, Elizabeth Glasier Pediatrics Aids Foundation etc) |  |
| Can you tell me what you know about the services which are provided through the mobile health clinics?   - When did this initiative start? - Why is this approach chosen? - Which health services are being delivered through mobile health facilities? - In which regions are these mobile health clinics operational? - Who is funding this initiative? - Are maternal health services among the services delivered by mobile health clinics? |  |
| Are there plans to include the mobile health clinics in the routine health service delivery especially for maternal health services?   - Dates/timelines, how to deliver, funding |  |
| Are there plans to scale-up the use of Mobile health Clinics to support maternal health services nationwide? |  |
| Has there been any discussion about strategies to improve utilization of maternal health services?   - What have been the proposed strategies? - Are proposed strategies being currently implemented? |  |
| What has been done to advocate and promote the use of mobile health clinics to deliver maternal health services:   - Any advocacy done - Any promotion done |  |
| How easy and difficult is it to use Mobile health Clinics to provide maternal health services? |  |
| How do you think we could make better use of mobile health clinics? |  |
| Do you have any concerns or question about using mobile health clinics to deliver maternal health services?   - Safety concerns - Logistics - Funding implications - Sustainability issues - Acceptability by different populations - Quality of services issues |  |
| In areas where utilization of maternal health services is still low, what do you think is the best way of improving uptake of services in those areas?   - Use of mobile health clinics - Use of outreach services - Use of campaigns - Health promotion and advocacy |  |
| How do you think we can best reach women who live in remote areas and provide them with maternal health services? |  |
| Do you see mobile health clinics as an opportunity to reach the remote hard to reach population with maternal health services? If yes, why? If no why not? |  |
| How can Mobile Health Clinics be made operational?   - What are the benefits? - What are the constraints? |  |
| Do you have any further suggestions? |  |

Thank you for the information and your time.
