# Supplementary material for: Policy makers’ perspective on the provision of maternal health services via mobile health clinics in Tanzania—Findings from key informant interviews
Source: PLoS One. 2018 Sep 7;13(9):e0203588. doi: 10.1371/journal.pone.0203588 (PMC6128610; doi:10.1371/journal.pone.0203588)
Supplement: S3 File — (DOCX) [file pone.0203588.s003.docx]

**Annex 1b**

**Semi-Structured Interview guide for Implementers**

| **Questions** | **Answers** |
| --- | --- |
| **Maternal Health Services Policies and Guidelines** |  |
| Are you aware of any policies that guide the provision of maternal health services in the country?   - If yes, which are they, and, what are the key elements of those? Policies? |  |
| In which area of maternal health services provision is your organization involved?   - What intervention, when did they start to get involved, and why and where are they providing these interventions? |  |
| What activities are currently being implemented to improve access of maternal health services?   - Mobile Health Clinics- Where? How often? Who is doing? Who is funding? - Community based interventions- Where? How often? Who is doing? Who is funding? |  |
| How successful to date has the effort to improve access of maternal health services in Tanzania especially for women living in remote areas?   - How do they define success? - What have been the challenges and opportunities? |  |
| **Maternal health services provision**  **(***Now I would like us to move on and discuss about different platforms that are used to provide maternal health services in Tanzania***).** |  |
| For how long has your organization being involved in providing maternal health services?  When did they start/ What pushed the decision to be involved in this area? |  |
| When and why did your organization start to provide health services through mobile clinics? |  |
| What services are specifically being provided by the mobile clinic?   - Ask about maternal health services |  |
| Where does your organization get funding to support the mobile clinics? |  |
| What has been your approach and experience in implementing mobile health clinics in the country? |  |
| How best can we reach women who live in remote areas with maternal health services? |  |
| **Using Mobile Health Clinics to provide maternal health services** |  |
| In your opinion do you think mobile health clinics are ideal to deliver Maternal Health services? |  |
| When, where and how did your organization managed to start implementing mobile health clinics?   - Are there future plans for scaling up? |  |
| Do you think that Mobile Health Clinics can be an opportunity to reach the unreachable mothers with maternal health services?  If yes, Why/ and if no, why not? |  |
| What are the challenges involved in delivering maternal health services through the mobile health clinics?   - Logistics - Manpower - Funding |  |
| How do you think we could make better use of mobile health clinics? |  |
| What other platform apart from the traditional provision of maternal health services through static health facilities, do you think they can also be an opportunity to in making sure maternal health services are accessible to people in remote areas? |  |
| Do you have any further suggestions? |  |

Thank you for the information and your time.
