# Supplementary material for: Policy makers’ perspective on the provision of maternal health services via mobile health clinics in Tanzania—Findings from key informant interviews
Source: PLoS One. 2018 Sep 7;13(9):e0203588. doi: 10.1371/journal.pone.0203588 (PMC6128610; doi:10.1371/journal.pone.0203588)
Supplement: S4 File — (DOCX) [file pone.0203588.s004.docx]

### ANNEX 2: KEY INFORMANTS ‘INTERVIEWS INFORMATION SHEET AND CONSENT FORM

**Feasibility and practicability of providing maternal health services through mobile health clinics in Tanzania**

Background information

An increasing number of women lack access to cost effective maternal health interventions in Tanzania. This is hindering the effort towards reducing maternal mortality in the country. Mobile health clinics can be used as a platform to reach the women who would otherwise not access services with maternal health services. Recent studies suggest that mobile health clinics can provide an alternative portal into the health care systems for the medical disenfranchised, that is providing people who are otherwise outside the reach and mainstream of health care due to simple issues like location and remoteness. In response to this the Tanzanian government through development partners is currently implementing delivery of maternal health services through this portal.

This study aims to explore the feasibility and practicability of providing maternal health services through mobile clinics. Ideally, the mobile health vans which are equipped with all necessary equipment’s and manpower needed to offer all maternal health services goes through the remote villages and put a station and offer services. In some cases they also offer delivery services when it happens that there is a need. Acknowledging that providing maternal health services out of the health facility setting is not the same as providing them in the usual health facility settings, understanding practicalities of implementation of this portal of service delivery is necessary. Furthermore is important to understand that not many mobile health services in the country have been used to provide other reproductive health interventions like family planning, immunization, and screening for STDs among other health interventions, however, their application in maternal health services may be different from those.

This study is part of the requirement for the fulfillment of the PhD studies of Dr. Nyasule Neke, who is currently enrolled at the Duisburg-Essen University, and, she is also working with the National Institute for Medical Research in Mwanza Centre. We have requested permission to conduct this study by the Medical Research Coordinating Committee of the Tanzanian government and the Duisburg-Essen University of the Germany.

Procedures

In order to know your views regarding the practicability and feasibility of using this portal to deliver maternal health services we will ask you to participate in an interview with one of our research team. This interview should take no longer than I hour and will be conducted in a private place. You are allowed to ask any questions regarding the study, and after you have had all your questions answered and you are satisfied with the response and you understand what you will have to do, we will request you to sign, or put your thumbprint on this consent form. The researcher will ask for your permission to record the interview. The interview will take a style of one on one discussion, and it will be casual and you will be encouraged to talk freely about anything you feel that is related to the questions about the provision of maternal health services through mobile health clinics.

Study Duration

We are planning to conduct the interview over a period of one month; however, you will only be interviewed once.

Risks, Stress, or Discomfort

There are no risks associated with your participation to this study. However, when it happen that you feel that you have suffered any harm as a result of your participation in this study kindly feel free to contact the person whose name is written below the end of this form.

Benefits

It is hoped that the information gained from the study will be useful in improving delivery of maternal health services through the mobile health clinics in Tanzania.

Participation

We hope that you will be available and willing to participate in this research. However, feel free not to participate in case you do not wish to participate in the interview. Additionally, you can withdraw from this research project at any time.

Confidentiality

Kindly lest assured that your contact details will be confidential and will only be shared within the staff involved in the study.

If you have any questions

If you have any questions about what we are doing or about the study in general, please contact Dr Nyasule Neke (Tel +255 717 816673). The project office is in the NIMR Mwanza Centre in Isamilo, Mwanza. The postal address and telephone number of NIMR is given at the top of this form.

### What you are required to do

If you are willing and agree to participate in this research, please sign below.

Subjects Statement and Signature

The study described above has been explained to me and I have been given a chance to ask questions. Moreover, I have received information and answers that made me understood the overall and specific aims of this study. I am hereby consenting to participate in this study by providing the answers to the questions according to my knowledge and ability. I am also aware that there is a possibility of asking further questions in the future regarding the study, and, I have been explained how that can be done together with the availability of the contacts details of the study corresponding personnel in this form. I will also remain with the signed version of this form.

## Participant’s Signature/ thumbprint

| Signature or Thumbprint |  | Date |  |
| --- | --- | --- | --- |
| Name in Capital Letters |  | | |

Witness’s signature (if participant is illiterate)

| Signature |  | Signature date |  |
| --- | --- | --- | --- |
| Name in Capital Letters |  | | |

Put a mark inside the box if participant is illiterate and does not want a witness

## Signature of researcher obtaining consent

| Signature |  | Signature date |  |
| --- | --- | --- | --- |
| Name in Capital Letters |  | | |

Research Team Addresses

1. Universität of Duisburg-Essen, Fachbereich Wirtschaftswissenschaften, Thea-Leymann-Str. 9, D-45127 Essen, Germany. Tel.: +49 (201) 183-4075, Fax: +49 (201) 183-4073
2. National Institute for Medical Research (NIMR) P.O. Box 11936, Isamilo Street Mwanza, Tanzania. Tel: +255-28-2500019, Fax: +255-28-2542162.
